# Microbial Extracellular Polymeric Substances: Ecological Function and Impact on Soil Aggregation

**DOI:** 10.3389/fmicb.2018.01636

**Published:** 2018-07-23

**Authors:** Ohana Y. A. Costa, Jos M. Raaijmakers, Eiko E. Kuramae

**Affiliations:** ^1^Department of Microbial Ecology, Netherlands Institute of Ecology (NIOO-KNAW), Wageningen, Netherlands; ^2^Institute of Biology, Leiden University, Leiden, Netherlands

**Keywords:** EPS production, microorganisms, biosynthesis, ecological functions, soil aggregation

## Abstract

A wide range of microorganisms produce extracellular polymeric substances (EPS), highly hydrated polymers that are mainly composed of polysaccharides, proteins, and DNA. EPS are fundamental for microbial life and provide an ideal environment for chemical reactions, nutrient entrapment, and protection against environmental stresses such as salinity and drought. Microbial EPS can enhance the aggregation of soil particles and benefit plants by maintaining the moisture of the environment and trapping nutrients. In addition, EPS have unique characteristics, such as biocompatibility, gelling, and thickening capabilities, with industrial applications. However, despite decades of research on the industrial potential of EPS, only a few polymers are widely used in different areas, especially in agriculture. This review provides an overview of current knowledge on the ecological functions of microbial EPSs and their application in agricultural soils to improve soil particle aggregation, an important factor for soil structure, health, and fertility.

## Introduction

Extracellular polymeric substances (EPS) are polymers biosynthesized by several strains of microorganisms. Composed mainly of polysaccharides, proteins, and DNA, the production of these slimes is triggered primarily by environmental signals. Since their biosynthesis is energetically expensive, they should generate some kind of advantage to the producer microorganism (Flemming and Wingender, 2010). Therefore, EPS production and functions have been studied for decades.

The polysaccharides are the most studied components of EPS. The investigation of EPS from numerous strains of microorganisms has demonstrated that the polysaccharides in these biopolymers vary immensely in composition and structure. They can be composed by one or many structural units, and the arrangement of these units is also exclusive for each different kind of EPS (Roca et al., 2015). Aside from the carbohydrates, recently the interest in the structural proteins, enzymes, and extracellular DNA (exDNA) has also been increasing. The analysis of exDNA present in the EPS of a variety of strains has shown that the DNA is not innocuous, but can be a source of genetic exchange, signaling, attachment, and moreover a very important structural component (Flemming and Wingender, 2010).

Besides the diversity of structures, EPS vary in their functions. A significant number of functions has been attributed to EPS, most of them related to protection. The matrix produced by EPS around microbial cells has the capability of shielding them against antimicrobial compounds and heavy metals; EPS matrix can also retain water, protecting microbes and the environment against drought. In addition, other functions, such as adhesion, communication with other microbes and plants, antioxidant, aggregation, carbon storage, and entrapment of nutrients have also been reported (Wingender et al., 1999b; Vardharajula and Ali, 2015; Wang et al., 2015).

One of the roles of the EPS matrix that has been explored for decades is the capacity to aggregate soil particles, a function that is important for soil structure, health, and fertility. Since EPS have a slimy texture and ionic charges, it can act like a glue, getting attached to clay and ions, holding solid particles together (Chenu, 1995). On the other hand, as stated before, EPS structures are variable; therefore, their application efficiency in soils will vary accordingly. These polymers that are studied and produced in laboratorial conditions can be applied to soils for improvement of soil structure, fertility, and quality. In this review, we collate and synthesize the available information on the ecological functions of microbial EPS and their application on soil particle aggregation. The information on EPS composition, biosynthesis, and factors affecting EPS production have been comprehensively described before (Wingender et al., 1999b; Flemming and Wingender, 2010; Sheng et al., 2010; More et al., 2014; Flemming et al., 2016; Nouha et al., 2017) and will be not described here.

## Ecological Functions

Initially, EPS were used as an abbreviation for “extracellular polysaccharides,” “exopolymers,” or “exopolysaccharides.” EPS can be produced by bacteria, cyanobacteria, microalgae (Parikh and Madamwar, 2006; Boonchai et al., 2014), yeasts (Pavlova and Grigorova, 1999), fungi (Hwang et al., 2004; Elisashvili et al., 2009), and protists (Jain et al., 2005; Lee Chang et al., 2014). EPS are biosynthetic polymers composed mainly of polysaccharides, structural proteins, enzymes, nucleic acids, lipids, and other compounds such as humic acids (Wingender et al., 1999a,b; Flemming and Wingender, 2010).

**FIGURE 1:**
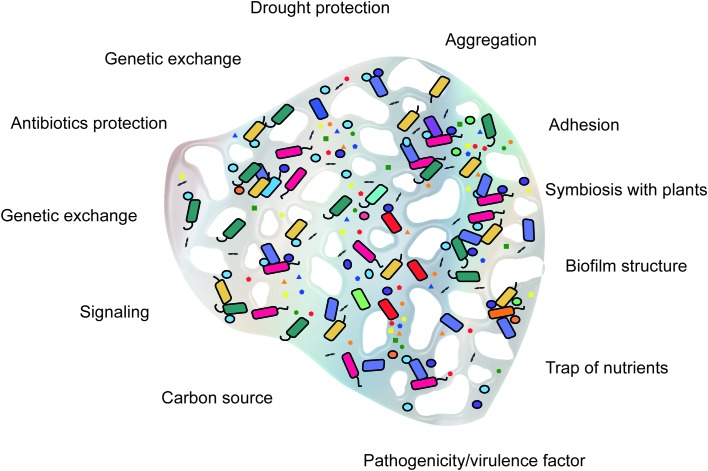
Conceptual framework of the functions of microbial extracellular polymeric substances (EPS) in soil.

Extracellular polymeric substances biosynthesis is an energy-demanding process. Therefore, their production requires selective advantages in the environment of the producing microorganism. In laboratory cultures, the production of EPS does not impact cell viability or growth and thus appears not to be essential for survival. However, in natural environments, most microorganisms live in aggregates, such as flocs and biofilms, for which EPS are structurally and functionally essential (Wingender et al., 1999b). Most of the functions attributed to EPS are related to protection of the producing microorganism. Diverse variations in abiotic conditions such as drought, temperature, pH, and salinity can trigger the production of EPS as a response to environmental stresses (Wingender et al., 1999b; Kumar et al., 2007; Vardharajula and Ali, 2015). The functions of EPS are summarized in **Figure 1**.

### Functions of EPS in Interactions With Other Microorganisms and Environment

#### Adhesion/Cohesion/Genetic Material Transfer

Extracellular polymeric substances are responsible for the cohesion of microorganisms and adhesion of biofilms to surfaces, influencing spatial organization, allowing interactions among microorganisms, and acting as adhesives between cells (Wolfaardt et al., 1999). These functions are important for the establishment and biological activities of biofilms and flocs. The polymers mechanically stabilize the microbial aggregates *via* several types of interactions between the macromolecules, including dispersion forces, electrostatic interactions, and hydrogen bonds. The resultant formation of a gel-like tridimensional structure around the cells allows the microorganisms to be retained near each other to establish stable consortia (Flemming et al., 2000). For example, EPS of *Sphingomonas paucimobilis* have surface-active properties that promote and enhance attachment *via* the formation of polymeric bridges (Azeredo and Oliveira, 2000). The quantity of EPS can also influence cell adhesion, as demonstrated by Tsuneda et al. (2003). For the 27 bacterial strains evaluated, small quantities of EPS inhibited cell adhesion by electrostatic forces, whereas large amounts enhanced adhesion *via* interactions between functional groups in the EPS, such as uronic acids and acetyl groups. The nature of the interactions between the functional groups in EPS, however, is unknown. In addition, the matrix formed by EPS can facilitate chemical communication and even influence predator–prey interactions (Flemming et al., 2007). Joubert et al. (2006) observed that ciliated protists pred feeding on planktonic cells and the EPS matrix rather than on attached and biofilm-derived cells. In addition, the presence of protists appeared to enhance yeast metabolic activity in the biofilm.

Together with different protein adhesins, EPS are believed to be involved in the initial steps of microbial adhesion to surfaces. For instance, the polysaccharide produced by *Caulobacter crescentus*, called holdfast, is crucial for the initial surface attachment, together with other cellular structures (Entcheva-Dimitrov and Spormann, 2004; Wan et al., 2013). However, the characteristics of each polymer are defined by their composition, as adhesiveness depends heavily on chain conformation, internal substituents, and internal/external interactions (Berne et al., 2015). Therefore, the extent to which the type of polymer contributes to the adhesive properties of bacterial cells remains to be determined.

In addition to polysaccharides, exDNA seems also to be responsible for the adhesive properties of some EPS. Although the functions of exDNA have not been completely elucidated, studies have shown that it is responsible for the cohesion and structure of certain EPS and plays a role in adhesion to surfaces and signaling (Okshevsky and Meyer, 2013). Released by autolysis or active secretion by microorganisms, exDNA is likely an important structural component of *Staphylococcus aureus*, *Pseudomonas aeruginosa*, and *Ralstonia solanacearum* biofilms (Whitchurch et al., 2002; Minh Tran et al., 2016); however, it is not essential in biofilms produced by *Streptococcus epidermidis* (Flemming and Wingender, 2010). This conclusion is based on the fact that treatment with DN*ase* *I* inhibits biofilm formation and detachment of preformed biofilms by *S. aureus* but not *S. epidermidis* (Izano et al., 2007).

Enhancement of genetic material transfer between microorganisms is another property of extracellular polymers. ExDNA of different origins is an important EPS component in biofilms, where microorganisms are surrounded by an EPS matrix. Although studies in this area are scarce, the rates of natural transformation and conjugation of bacteria appear to be higher within biofilms. Bae et al. (2014) demonstrated that *Campylobacter jejuni* transfers antibiotic resistance genes by natural transformation more frequently in biofilms than in planktonic cells. Other studies have shown that biofilm age and DNA concentration influence the frequency of transformation events, whereas a high density of planktonic cells inhibits transformation in biofilms (Hendrickx et al., 2003). Moreover, the number of events observed can depend greatly on the technique used to detect conjugative gene transfer in biofilms. For instance, Hausner and Wuertz (1999) detected 1000-fold higher conjugation rates using confocal laser scanning microscopy (CLSM) than by classic plating techniques. It has been suggested that exDNA fractions can be used in environmental studies as an alternative method for microbial activity measurement. However, exDNA fraction separation and evaluation in complex samples, such as soils, has yet to be improved (Nagler et al., 2018). Estimates of microbial community composition can be influenced by the presence of exDNA (Carini et al., 2016).

### EPS in Microbe–Host Interactions

#### Symbiosis

Extracellular polymeric substances play an important role in the establishment of symbiosis between nitrogen-fixing rhizobia and plants. Rhizobial surface polysaccharides are fundamental for nodule formation by some legumes, although the underlying mechanisms are not yet fully resolved. For example, to invade alfalfa nodules and establish successful symbiosis, *Sinorhizobium meliloti* Rm1021 must produce succinoglycan (Cheng and Walker, 1998). Mutants that do not synthesize succinoglycan, produce modified polymers or overproduce EPS, reduce the capacity of *S. meliloti* Rm 1021 to infect and establish symbiosis. Although capable of producing nodules, *Rhizobium leguminosarum* biovar *viciae* glucomannan (*gmsA*) mutants are strongly outcompeted by wild-type bacteria in mixed inoculations of *Pisum sativum* (Williams et al., 2008). The interaction between the EPS of *Mesorhizobium loti* strain R7A and *Lotus japonicus* was recently shown to be mediated by a receptor expressed by the plant. *L. japonicus* produces a receptor (EPR3) that binds to and permits infection by only bacteria that produce EPS with a specific structure; mutants with truncated EPS are less successful in infection (Kawaharada et al., 2015). The expression of this receptor demonstrates that the plant is capable of recognizing the structure of EPS produced by rhizobia.

#### EPS as Pathogenicity/Virulence Factors

For some bacteria, polymers function as pathogenicity and virulence factors. For example, the high virulence of *Erwinia amylovora* is a result of the production of amylovoran and levan. Both polymers contribute to the pathogenesis of the bacteria, and the absence of either amylovoran or levan dramatically decreases plant colonization (Koczan et al., 2009). In addition, EPS can serve as a mechanical barrier between bacteria and plant defense compounds by decreasing the diffusion rates of these compounds. For example, the polymers of *Pseudomonas syringae* pv. *phaseolicola* and *S. meliloti* protect the bacteria against reactive oxygen species (ROS) produced by the plant host during infection, thereby decreasing oxidative stress (Király et al., 1997; Lehman and Long, 2013). *S. meliloti* mutants overproducing EPS protect polymer-deficient mutants against H_2_O_2_ (Lehman and Long, 2013). Alginate, the EPS produced by *P. aeruginosa*, a human opportunistic pathogen, protects the bacteria against the inflammatory process of the host, avoiding free radicals, antibodies, and phagocytosis and thereby aggravating the prognosis of patients infected by *P. aeruginosa* (Ryder et al., 2007). Although it is known that EPS may act as an antioxidant, less is known about the chemical mechanism of protection against ROS.

### EPS and Nutrition

#### Carbon Reserves

Extracellular polymeric substances produced by microorganisms might act as carbon reserves, but few studies have investigated the role of EPS in nutrition or cross-feeding between organisms. Since EPS are generally complex molecules, their complete degradation would require a wide range of different enzymes (Flemming and Wingender, 2010). Rhizobium NZP 2037 can use its own poly-β-hydroxybutyrate (PHB) and EPS as sole carbon sources for survival in carbon-restriction situations (Patel and Gerson, 1974). However, EPS is a higher potential carbon source than PHB. Stable isotope probing (SIP) is a powerful strategy for detecting microorganisms that can degrade polymers. Wang et al. (2015) labeled the EPS of *Beijerinckia indica* and observed that the polymer was assimilated by bacteria with low identities to known species, particularly members of the phylum *Planctomycetes*. In addition, the authors isolated bacteria that used the EPS as a sole carbon source, demonstrating the potential utility of these polymers for isolating new microbial species.

#### Nutrient Trap

In addition to supplying carbon, EPS can accumulate other nutrients and molecules. The retention of extracellular enzymes in the EPS matrix promotes the formation of an extracellular digestion system that captures compounds from the water phase and permits their use as nutrient and energy sources (Flemming and Wingender, 2010). Many studies have investigated the adsorption of metal ions by EPS for heavy-metal remediation and recovery of polluted environments. In soils, microbial EPS can sorb, bind or entrap many soluble and insoluble metal species, as well as clay minerals, colloids, and oxides, which also have metal binding properties (Gadd, 2009). In addition, EPS can form networks with other EPS (Etemadi et al., 2003). Most of the studied EPS are negatively charged, due to the dominance of carboxyl and hydroxyl functional groups, in different proportions depending on EPS composition (Ding et al., 2018). The main factors influencing metal biosorption by EPS are related to the binding sites or their chemical nature, such as pH, metal content, ionic strength, surface properties, as well as EPS molecular weight and degree of branching (Guibaud et al., 2003; Fukushi, 2012). For instance, it was demonstrated that the structural conformation of xanthan affects Cu–xanthan bond strength. Xanthan presented an unusual sorption behavior as Cu sorption decreased at increasing pH values between 3.5 and 5.5. In this condition, sorption should increase, due to less competition with protons; however, the results can be explained by the conformational changes of xanthan (Causse et al., 2016). In other studies, the potential of biosorption of a variety of metals by several EPS was already evaluated. The EPS of *Paenibacillus jamilae* adsorbs multiple heavy metals (Pb, Cd, Co, Ni, Zn, and Cu) with stronger interaction with Pb, a maximum binding capacity of 303.03 mg/g, 10-fold higher than the binding capacities for other metals (Morillo Pérez et al., 2008). The polymers produced by *Anabaena variabilis* and *Nostoc muscorum* possess similar affinities for Cu, Cd, Co, Zn, and Ni, with the highest affinity for Cu and the lowest for Ni. Both bacterial EPS are promising for the removal of toxic heavy metals from polluted water (El-Naggar et al., 2008). Similarly, the EPS of *Cyanothece* sp. CCY 0110 is capable of removing Cu, Pb, and Cd from aqueous solutions (Mota et al., 2016). The EPS of *Pseudomonas* sp. CU-1 has a high Cu-binding capacity and thus, protect bacterial cells against this metal ion (Lau et al., 2005). The EPS of *Azotobacter chroococcum* XU1 is capable of absorbing, from an aqueous solution, 40.48 and 47.87% of Pb and Hg, respectively (Rasulov et al., 2013). Interestingly, Raliya et al. (2014) used ZnO nanoparticles to induced higher EPS production from *Bacillus subtilis* strain JCT1, which was later applied in a sandy soil and improved aggregation in 33–83%.

### EPS in Protection Against Abiotic and Biotic Stresses

#### Drought Protection

Extracellular polymeric substances production can confer advantages to microorganisms in environments under drought stress. A high water-holding capacity was observed for an EPS produced by a *Pseudomonas* strain isolated from soil; this EPS can hold several times its weight in water. When added to a sandy soil, the EPS altered its moisture by allowing the amended soil to hold more water than unamended soil (Roberson and Firestone, 1992). According to the authors, the EPS protected the bacteria against desiccation by acting like a protective sponge, thereby giving the bacteria time to make metabolic adjustments. This polymer exhibits significant structural modifications during desiccation and may be an important protection factor, trapping a reservoir of water and nutrients for bacterial survival (Roberson et al., 1993). Cyanobacteria isolated from arid regions, such as *Nostoc calcicola* (Bhatnagar et al., 2014) and *Phormidium* 94a (Vicente-García et al., 2004), are also capable of producing EPS, which may represent a strategy for water/nutrient retention and survival.

#### Salt Tolerance

Some studies have revealed that microbial polymers are involved in tolerance to salt stress, not only for the producer microorganisms but also for the associated plants. The production of polymer by NaCl-tolerant isolates can decrease Na uptake by plants by trapping and decreasing the amount of ions available (Upadhyay et al., 2011). Therefore, the polymer prevents nutrient imbalance and osmotic stress, which can promote survival of the microorganisms and benefit the plant. *S. meliloti* strain EFBI cells severely reduce EPS production when inoculated in culture medium with low salt concentration. Since this strain was isolated from the nodules of a plant growing in a salt marsh with a salinity level of 0.3 M, a lower amount of salt can be considered a stressful condition. However, the relevance of this EPS for survival and symbiosis was not further studied (Lloret et al., 1998).

#### Protection Against Low/High Temperatures

The production of EPS at low temperatures is an important factor in the cryoprotection of sea-ice organisms as well as a natural adaptation to low temperatures and high salinities. High concentrations of EPS have been observed in samples collected from Arctic sea ice; the EPS shields microorganisms against the severe environmental conditions during the winter season (Krembs et al., 2002; Caruso et al., 2018). In addition, EPS alter the microstructure and desalination of growing ice, consequently improving microbial habitability and survivability (Krembs et al., 2011).

Extracellular polymeric substances can be a protection factor for thermophilic bacteria by shielding microorganisms from very high temperatures. The polymers produced by *Bacillus* sp. strain B3-72 and *Geobacillus tepidamans* V264 are not easily dissolved at high temperatures (Nicolaus et al., 2000; Kambourova et al., 2009). A few studies (Manca et al., 1996; Nicolaus et al., 2000; Nicolaus et al., 2004) have evaluated EPS production by thermophilic bacteria and archaea for potential applications of these polymers in industry and the recovery of polluted environments. However, the structure and the ecological function of these slimes remain to be established.

#### Protection Against Antimicrobials

The matrix that surrounds microorganisms in biofilms plays an important role in decreased susceptibility to antimicrobials. In general, biofilm matrices possess a negative charge and therefore bind positively charged compounds, protecting the innermost cells from contact. In addition, electrostatic repulsion can reduce the diffusion rates of negatively charged antimicrobials through the biofilm (Everett and Rumbaugh, 2015). Many studies have tested the inhibitory potential of bacterial EPS against antimicrobial compounds, particularly for clinically important bacterial strains. A few studies have demonstrated that the slime produced by *Staphylococcus* sp. is an effective antagonist to vancomycin, perfloxacin, and teicoplanin, acting as a barrier to the compounds or even interfering with their action in the cell membrane (Farber et al., 1990; Souli and Giamarellou, 1998). The EPS produced by *Acinetobacter baumannii* is also protective against tobramycin exposure and is effective regardless of the bacterial species exposed. By contrast, the polymer from *S. aureus* has no protective effect against tobramycin (Davenport et al., 2014). EPS can also protect microorganisms against disinfection agents. Alginate produced by *P. aeruginosa* enhances bacterial survival in chlorinated water, and removal of the slime eliminates bacterial chlorine resistance (Grobe et al., 2001).

The few EPS isolated thus far have a wide range of functions, but a huge diversity of polymers produced by microorganisms with different functions awaits exploration and discovery. The different functions already discovered are consequences of the diverse EPS structures and are connected to the benefits they can have when applied to soils. The production of EPS is not only an advantage to the microbes but also to the soil environment in general. The adhesiveness is important for gluing soil particles together; high water holding capacity protects microorganisms and plants against drought, as well as permits the diffusions of nutrients in the environment. EPS production also influences and is influenced by interactions between plants and microorganisms, thereby increasing the availability of nutrients as a whole, promoting plant and microbial growth. In the next section, we summarize how the currently known EPS are applied to agricultural soils and their benefits for soil aggregation.

## Application of Eps on Soil Aggregation

### Soil Aggregates and Microbial Communities

Aggregates are the basic units of soil structure and are composed of pores and solid material produced by rearrangement of particles, flocculation, and cementation. These units define the physical and mechanical properties of soil, such as water retention, water movement, aeration, and temperature, which in turn affect physical, chemical, and biological processes (Alami et al., 2000; Tang et al., 2011). Aggregates are important for the improvement of soil fertility, porosity, erodibility, and agronomic productivity by influencing plant germination and root growth (Dinel et al., 1992; Bronick and Lal, 2005). Aggregate formation involves numerous factors: vegetation, soil fauna, microorganisms, cations, and interactions between clay particles and organic matter (Kumar et al., 2013). The stability of aggregates depends on their internal cohesion, pore volume, connectivity, tortuosity, and pore-wall hydrophobicity (Chenu and Cosentino, 2011). A good soil structure, dependent on aggregation, is fundamental for sustaining agricultural productivity and environmental quality, sustainable use of soil, and agriculture (Amézketa, 1999).

**FIGURE 2:**
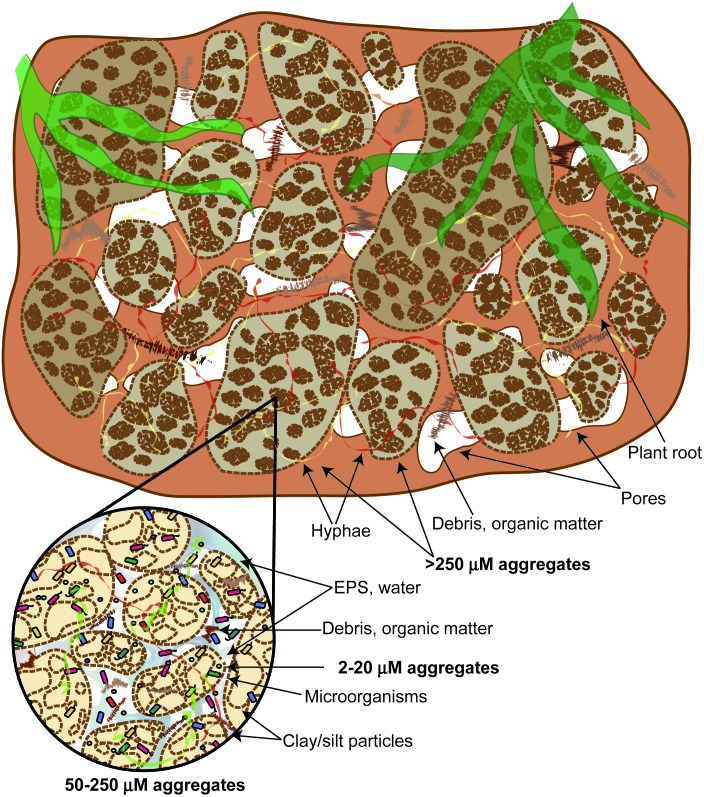
The hierarchical model of soil aggregate classification. Larger aggregates are composed of smaller units, which are formed from even smaller aggregates.

The hierarchical model for classifying soil aggregates suggests that larger aggregates are composed of smaller units, which are formed from even smaller aggregates (Tisdall and Oades, 1982; **Figure 2**). In persistent microaggregates (2–20 μm diameter), clay particles are united by inorganic amorphous binding agents such as aluminosilicates, oxides, humic substances, and soil polysaccharides associated with metal ions. These persistent microaggregates are bound together into larger microaggregates (20–250 μm diameter) by plant roots, root hairs, and fungal hyphae. Microaggregates are glued to each other by transient binding agents such as polysaccharides and polyuronides to form macroaggregates (>250 μm diameter). Aggregation is influenced by the soil microbial community, mineral and organic compounds, plant community composition, and past soil handling (Tisdall and Oades, 1982). Among the most important minerals involved in microaggregate formation are carbonates (CaCO_3_), Fe- and Al-(hydr)oxides, and clay minerals (Totsche et al., 2018). EPS are directly involved in the formation of organo-mineral associations in soil, affecting the composition of immobile and mobile organic matter, as well as the reactivity of minerals (Liu et al., 2013). EPS aggregate mineral particles by adsorbing onto mineral surfaces, creating connections between different types of minerals and enhancing their ability to retain water (Henao and Mazeau, 2009). Several studies investigated EPS-mineral adsorption, performing the visualization of the complexes using microscopy. For instance, Lin et al. (2016) demonstrated that electrostatic interactions are important in the interaction between the EPS of *Pseudomonas putida* X4 and minerals kaolinite, montmorillonite, and goethite. The EPS C, N, and P fractions had a higher adsorption capacity at a lower pH, due to protonation of some EPS groups. In addition, authors used CLSM to visualize the distribution of polysaccharides, proteins, and nucleic acids in mineral-EPS complexes. Liu et al. (2013) studied the adsorption of the EPS from *B. subtilis* 168 to goethite and observed that goethite-adsorbed EPS was enriched in EPS fractions that contained mainly lipids and proteins, but depleted in polysaccharides. The proteins adsorbed were rich in S, derived from S containing amino acids. On the other hand, pure EPS was dominated by proteins and polysaccharides, with low amount of lipids and nucleic acids.

For many decades, the microbial communities inside different classes of aggregates have been investigated using several techniques and experimental designs (Blaud et al., 2012, 2017; Davinic et al., 2012; Zhang et al., 2018). Many studies determined the microbial community inside the different aggregate sizes in different agriculture management systems (Sessitsch et al., 2001; Mummey and Stahl, 2004; Kravchenko et al., 2014); however, no studies evaluated the microbial community responsible for the aggregation. Studies on microbial effect on soil aggregations were limited to microbial isolated strains, albeit the role of microorganisms and their polysaccharides in soil aggregation have been studied for decades. Caesar-TonThat et al. (2007) used microaggregates (250–50 μm) from two agricultural ecosystems (40 years tillage and 9 years no tillage) to isolate bacteria and test their aggregation potential, as well as profiled both systems using fatty acid methyl ester (FAME). They observed that *Stenotrophomonas*, *Sphingobacterium*, *Bacillus*, and *Pseudomonas* species could stabilize and increase aggregate strength in artificial aggregates, and that these species were frequent in partially undisturbed soils. In other study, Caesar-Tonthat et al. (2014) investigated if soil aggregation and the culturable aggregating bacteria present in soils were influenced by different irrigation, tillage, and cropping systems. In the irrigated no tillage and conservation areas, higher proportion of soil aggregating bacteria were isolated (81, compared to ∼35). They were able to isolate 50 aggregating bacteria (from 1296 isolates), which were dominated again by *Pseudomonas* sp. and *Bacillus* sp. Interestingly, *Bacillus* and *Pseudomonas* are genus widely known to produce biofilms and EPS, which are involved in the stabilization of soil structure.

### Inoculation of EPS Producers in Soils

Microorganisms are fundamental for soil aggregation and stabilization. However, the influence of microorganisms on soil structure stabilization varies and depends on the microbial species, available substrates, and soil management (Beare et al., 1994; Umer and Rajab, 2012). Bacteria and fungi contribute to stabilization of soil structure by producing extracellular polymers and degrading aromatic humic materials that generate clay–metal–organic matter complexes (Umer and Rajab, 2012). Fungi also contribute by anchoring particles through hyphae, albeit with less persistence. The aggregating potentials of numerous bacterial and fungal strains have been tested, demonstrating that the effect of microbial pure cultures on soil aggregation is dependent on the microbial species. Therefore, different microbial slimes and EPS have been explored as aggregation-capable components in different types of soils, for the recovery of soil quality and fertility.

Among the bacterial EPS producers that are the most investigated for soil aggregation potential are strains of *Pseudomonas*, *Bacillus*, and *Paenibacillus*, genera easily grown in laboratorial conditions, producing high amounts of EPS. In addition, strains of *Streptomyces* and *Penicillium* showed a significant positive effect on soil loss and erodibility, after rainfall simulation (Gasperi-Mago and Troeh, 1979). *P. putida* strain GAP-P45 inoculation in soil increased aggregate stability in more than 50% in soils subjected to temperature, salt, and drought stresses. Under stress conditions, the strain produced more EPS, protecting the bacteria against water stress and contributing to soil structure (Vardharajula and Ali, 2015). An unidentified bacterium isolated from biological soil crusts (BSCs) from the Gurbantünggüt Desert stabilized sand surface, producing aggregation and slowing the soil water evaporation after only 8 days of inoculation. In addition, the EPS of the bacterium produced the conglutination of sand particles, as observed by scanning electron microscopy (HuiXia et al., 2007). Another isolate from Gurbantünggüt Desert, *Paenibacillus* KLBB0001 – a strong EPS producer – was inoculated in the desertic soil to improve the recovery of BSCs. After 1 year of field experiments, the strain stimulated the heterotrophic community in the soil and increased the numbers of bacteria, available nitrogen, and phosphorus. Microscope images of the inoculation area revealed a glue-like polymer connecting sand grains, confirming the presence of EPS (Wu et al., 2014). The studies showed the potential of the strains for the recovery of soil structure, especially under nutrient- and water-limited conditions.

Due to its high EPS production, *Bacillus* *amyloliquefaciens* strain HYD-B17, *B. licheniformis* strain HYTAPB18, and *B. subtilis* RMPB44 inoculation in soil improved aggregate stability in both the absence of stress and under drought stress conditions. For these strains, it was also observed a better aggregation effect with a larger bacterial population size, as well as an important role of larger incubation periods for EPS production and soil aggregation. All the strains produced more EPS under drought conditions, and strain HYD-B17 was the most efficient for aggregation among the strains studied. The differences in the performances of the strains could be explained by the different compositions of their EPS. The performance of the strains demonstrate that they are also interesting for inoculation in situations of abiotic stresses (Vardharajula and Ali, 2014). Strains of *Pseudomonas* and *Bacillus* were also important for the stabilization of sand on the beach and at the edge of a dune in the study of Forster (1979).

*Microbacterium arborescens* AGSB is another example of an EPS-producing strain that can be used for the recovery of soils; its inoculation produces strong binding in sandy soil. In addition, the bacteria produced better aggregation in a sandy soil than in agricultural and mine reject soils, showing that the effect of microbial inoculation varies according to the soil type (Godinho and Bhosle, 2009).

In addition to other bacterial genera, the inoculation of soil with cyanobacteria has long been proved to be beneficial to soil structure and parameters. These bacteria are important in the stabilization of soil surfaces, primarily because of EPS production. In arid environments, cyanobacteria are major components of BSCs. BSCs are microbial assemblages developed on the top soil of drylands (Malam Issa et al., 1999). They are integral components of arid and semi-arid ecosystems, which biological activities are important for soil fertility and reduction of erosion, influencing soil temperature, C and N content, hydrological dynamics, and plant germination (Chamizo et al., 2012; Rossi et al., 2017; Velasco Ayuso et al., 2017). Their main components are species of bacteria, microalgae, fungi, lichen, and mosses, but their specific composition is variable (Wu et al., 2014; Rossi et al., 2017). The use of cyanobacteria for recovery of drylands and BSCs will not be discussed in this review since the focus is in agriculture soils.

Cyanobacterization improves soil structure, fertility, and bioavailability of nutrients, benefits that are extended also to the subcrust. Recently, they have been investigated for improvement of quality of arable lands and treatment of degraded and desertified environments (Rossi and De Philippis, 2015). Characteristics such as stress tolerance, drought resistance, and oligotrophy make them optimal candidates, and their EPS improves soil stability and moisture content at the topsoil, stimulating soil biological activity (Guo et al., 2007). Cyanobacteria exert a mechanical effect on soil particles, as they produce a gluing mesh, binding soil particles with their EPS. They promote the formation of hard entangled superficial structures that improve the stability of semi-arid soil surfaces, protecting them from erosion. In addition, they play a significant role in water storage, because of the hygroscopic properties of the EPS (Mugnai et al., 2017). For instance, the inoculation of *N.* *muscorum* improved the aggregate stability of a poorly structured silt loam soil in a greenhouse experiment. In this study, the authors investigated the effect of the inoculation of *N. muscorum* on the microbial population, soil nutrient status, and fertility. The addition of the microorganism increased soil aggregation by an average of 18%, as well as increased soil total carbon by ∼60% and total N by more than 100%; it also increased microbial population numbers and the emergence of lettuce seedlings in more than 52% (Rogers and Burns, 1994). Another strain of the genus *Nostoc* caused a positive impact in the physical characteristics of poorly aggregated soils from Guquka (Eastern Cape, South Africa). A dense superficial network of cyanobacterial EPS filaments covered soil surface after 4 and 6 weeks of incubation. The improvement appeared a short while after incubation, and increased with time and cyanobacteria growth (Malam Issa et al., 2006). Other strains of cyanobacteria, such as *Oscillatoria*, *Lyngbya*, and *Schizothrix delicatissima* AMPL0116 also showed positive effects in soil structure, by improving soil hydrological responses to rainfall, soil particle connections, soil permeability, and water absorption (Mugnai et al., 2017; Sadeghi et al., 2017).

Inoculation of pure cultures of filamentous fungi is known to increase soil aggregation, however, with different effectiveness than that of bacteria. Fungi not only can produce EPS that bind soil particles together but also produce hyphae that can enmesh aggregates (Baldock, 2002). The presence of *Stachybotrys atra* increased the aggregation of fumigated Peorian loess soil. However, the fungus was only able to produce this effect in a situation of reduced microbial community, demonstrating its establishment as the dominant microorganism (McCalla et al., 1958). In the study of Aspiras et al. (1971), *Alternaria tenuis*, *S. atra*, *Aspergillus niger*, *Mucor hiemalis*, and the streptomycetes *Streptomyces purpurascens* and *S. coelicolor* promoted the stabilization of artificial soil particles from three different soils. The aggregation was a result of binding agents closely associated to the hyphae. Swaby (1949) tested the aggregation capacity of pure cultures of 101 bacteria, 5 yeasts, and 50 filamentous fungi, finding that fungi had the best results. Among the best fungi there were species of *Absidia*, *Mucor*, *Rhizopus*, *Chaetomium*, *Fusarium*, and *Aspergillus*. For bacteria, *Achromobacter*, *Bacillus*, and unclassified *Actinomycetes* had the best aggregation potentials. A saprophytic lignin decomposed evaluated by Caesar-Tonthat and Cochran (2000) was able to aggregate and stabilize sandy soil, producing 90% of water-stable aggregates. The fungus excreted insoluble extracellular compounds that acted as binding agents, forming a fibrillary network observed in soil micrographs. *A.* *chroococcum*, *Lipomyces* *starkey*, and strains of *Pseudomonas* sp. and *M.* *hiemalis* were also able to promote soil stabilization (Lynch, 1981).

In addition of pure cultures, a combination of microorganisms can be an interesting option for soil inoculation. However, few studies investigated the addition of microbial consortia for improvement of soil aggregation. Nonetheless, when complex mixed cultures of microorganisms are inoculated (Swaby, 1949) in particles, aggregation is maximized as a result of interactions between different strains. Different species have different EPS properties; furthermore, EPS can have a complementary effect when associated with other EPS and other aggregating factors, such as EPS-coated fungal hyphae, resulting in greater adherence of soil particles compared to only physical involvement by the hyphae (Aspiras et al., 1971). Moreover, the combination of organic fertilizers with microbial inoculants can strengthen microbial aggregation effects by enhancing EPS production, consequently improving soil structure, function, and quality (Rashid et al., 2016).

### Plant Inoculation With EPS Producers

Plant inoculation with plant growth promoter rhizobacteria (PGPR) and arbuscular mycorrhizal fungi (AMF) is a very important agricultural practice. Microorganisms establish interactions with plants, promoting plant growth, which stimulates the microbial community with the production of exudates. The organic carbon released by plant roots stimulates the growth of the microbial communities in the rhizosphere, which in turn, produce mucilaginous EPS, promoting soil aggregation and increasing root adhering soil (RAS). RAS aggregation forms the immediate environment where plants take up water and nutrients for their development. The inoculation of plants with beneficial microbes can, in addition, increase the availability of nutrients, such as N, P, K, and iron (Rashid et al., 2016).

Among the best and most investigated bacterial candidates for plant inoculation are strains of *Bacillus*, *Pseudomonas*, *Rhizobium*, and *Pantoea*, all known EPS producers and plant growth promoters. These strains can be inoculated directly in soil, or in seedlings, where they will also be beneficial for crop yield (Cipriano et al., 2016). The production of EPS in the rhizosphere of plants protects the environment against drying and fluctuations in the water potential, increasing nutrient uptake by plants and promoting plant growth. It protects seedlings from drought stress and stimulates root exudates. The improvement in aggregation and soil structure improves the growth of seedlings, because it promotes an efficient uptake of nutrients and water (Alami et al., 2000; Bezzate et al., 2000; Sandhya et al., 2009). Several studies have evaluated the effect of PGPR and AMF; however, there was no focus on soil aggregation, since the most of the focus was on the plant growth.

*Rhizobium* strain KYGT207, which was isolated from an arid Algerian soil, is a wheat (*Triticum durum* L.) growth promoter and EPS-producing bacterium with significant soil structure-improving capacity. Inoculation of the strain on wheat increased the root-adhering soil dry mass/root dry mass ratio by 137% and enhanced the percentage of water-stable aggregates due to reduction of soil water stress by the EPS (Kaci et al., 2005). Equally significant are the effects of *Pantoea agglomerans* NAS206 and its polymer on the rhizosphere of wheat and on soil aggregation. The strain can colonize the wheat rhizosphere, causing significant aggregation and stabilization of root-adhering soil. It also increased aggregate mean diameter weight, formation of water-stable aggregates (diameter >0.2 mm), and RAS macroporosity. Thus, *Pantoea agglomerans* NAS206 is an interesting candidate for inoculation, since it can play an important role in regulating water content in the rhizosphere of wheat and in improving soil aggregation (Amellal et al., 1998, 1999).

The levan produced by *Paenibacillus polymyxa* CF43 also has notable effects on the aggregation of soil adhering to wheat roots (Bezzate et al., 2000). The authors tested the role of levan in aggregation of soil adhering to wheat roots by producing a mutant strain. In comparison with the mutant, the wild-type EPS producing strain increased the mass of RAS, demonstrating the influence of the EPS in aggregation and suggesting that the production of levan is the main mechanism involved in the improvement of the RAS structuration.

The role of EPS in soil aggregation has also been evaluated under the application of different environmental stresses. Inoculation of chickpea plants (*Cicer arietinum* var. CM-98) with the EPS-producing strains *Halomonas variabilis* HT1 and *Planococcus rifietoensis* RT4 protected the plants from salinity, promoted plant growth, and improved soil aggregation in more than 75% under elevated salt stress. These results demonstrated that both bacteria can be applied to enhance plant growth and soil fertility under salinity (Qurashi and Sabri, 2012). In another study, the EPS-producing *Rhizobium* YAS-34 positively affected soil aggregation and water and nitrogen uptake by sunflower plants under normal and water stress conditions. It increased RAS in up to 100%. The strain acted as a plant growth promoter, increasing shoot and root biomass and also soil macropore volume. These effects were attributed to EPS production, which increased soil water holding capacity (WHC) and reduced water loss (Alami et al., 2000). The strains of *Bacillus* and *Aeromonas* evaluated by Ashraf et al. (2004) increased the aggregation around roots of wheat in a moderate saline soil, restricting Na uptake by plants and promoting plant growth.

The effects of plant inoculation of several fungi have also been extensively evaluated, again with more focus on plant growth promotion than in rhizosphere soil aggregation. The mechanisms involved in the aggregate stabilization by fungi are entanglement of the soil particles by hyphae as well as the production of EPS. AMF also produce glomalin, a glycoprotein that acts as a glue (Kohler et al., 2006). Forster and Nicolson (1981) examined the effects of the interactions among grass (*Agropyron junceiforme*) and microorganisms (*Penicillium* sp. and *Glomus* *fasciculatus*) in the aggregation of sand from an embryo dune. Experiments showed that the addition of selected microorganisms increased both plant growth and soil aggregation. Even though roots alone affected sand aggregation, the best results were due to the association of microorganism inoculation and plants.

The mycorrhizal inoculation of *Olea europaea* and *Rhamnus lycioides* with *Glomus* *intraradices* showed beneficial effects for rhizosphere aggregation. Together with the addition of composted residue, AMF inoculation increased rhizosphere aggregation in comparison with non-rhizosphere soil by 1.8-fold (Caravaca et al., 2002). The effects of the inoculation of *G. intraradices* and *Pseudomonas mendocina* were evaluated by Kohler et al. (2006) in lettuce. The inoculation of both strains increased the percentage of water-soluble carbohydrates and stable aggregates. *P. mendocina* also had a positive effect on soil enzymatic activities, such as dehydrogenase and phosphatase. The combination of *P. mendocina* with inorganic fertilization increased stable aggregates in 84% compared to the control.

Inoculated microorganisms can have a significant effect on soil properties and quality by interacting with natural microorganisms in the rhizosphere, in addition to the improvement of plant productivity. Good soil structure and aggregate formation are important for controlling germination and root growth. Microbial inoculants have been studied for decades, but there is still a need for the enhancement of microbial growth conditions, for the production of high quality inoculants, with higher biomass and EPS production. Therefore, strains will be able to have an optimal performance in field conditions, with efficient colonization and dominance over the native microbial community.

### Addition of Pure EPS to Soil

Several studies link microbial products to soil aggregate stability. Polysaccharides are involved in the maintenance of soil structure, even though they are not the primary aggregating agents. Other molecules, such as humic acids, are also responsible for soil structure. The treatment of natural and synthetic soil aggregates with various chemical substances, such as periodate and tetraborate frequently does not result in a consistent pattern, demonstrating that polysaccharides are important, but more than one single substance are the main factors sustaining soil aggregates (Mehta et al., 1960; Sparling and Cheshire, 1985). Angers and Mehuys (1989) observed that the correlation between aggregate mean weight and carbohydrate content suggested that at least part of the water-stable aggregation was related to carbohydrates in soils cultivated with different crops. Treatment of the soil with sodium periodate prior to wet sieving confirmed partial involvement of carbohydrates in the stabilization of aggregates.

The resistance of the biopolymers to degradation may be related to their importance for the soil structure. The greater the resistance, the longer is the EPS persistence in soils. The association of polymers with metal ions and colloids, such as clay may also influence the degradation rates of polymers, because of their influence in enzymatic activity. Since the addition of polymers to soil started to be investigated, it has been demonstrated that the binding power of plant and microbial polysaccharides is variable. However, characteristics of the soil such as pH also influence the action of polysaccharides, because the charges of molecules are essential for binding particles (Martin, 1971). Some characteristics of polysaccharides that influence their binding activity are linear structure, length and flexible nature, that allow the formation of Van der Waals forces; large number of OH for hydrogen bonding and presence of acyl groups, allowing ionic binding to clays (Martin, 1971).

The effects of many different EPS produced by fungi and bacteria were already tested as soil aggregating agents. The direct application of polymers in soil can be an alternative to the inoculation of microorganisms. The aggregating potential of the EPS of *B. subtilis*, *Leuconostoc dextranicum*, and *L. mesenteroides* were evaluated by Geoghegan and Brian (1948). The different EPS had a significant result in soil aggregation tested by wet sieving, and even small amounts of levan (0.125–0.05%) were able to stabilize aggregates. The EPS of *Chromobacterium violaceum* had also an interesting effect in soil, being more resistant to degradation than a variety of plant polysaccharides. It exhibited the best binding performance among all polysaccharides tested, improving the hydraulic conductivity of a soil with neutral pH (Martin and Richards, 1963).

Some EPS molecules have a very high WHC. A xanthan tested by Chenu and Roberson (1996) demonstrated a WHC of 15 times its weight. The dextran tested in the same study had a lower WHC, due to differences in structure. For both EPS, diffusion of glucose was tested, and it was observed that diffusion rates were slower than in water. A high WHC of EPS can protects microorganisms, soil and plants against drought stress, promoting hydrating conditions and bridging among soil particles and clay. In addition, the nutrients are still able to diffuse until the microorganisms during low water potentials, maintain physiological functions even during dry periods. The EPS of a *Pseudomonas* strain isolated from soil can also hold several times its weight in water. When added to a sandy soil, the EPS altered its moisture by allowing the amended soil to hold more water than unamended soil. The addition of a small amount of EPS increased the amount of water held by the sand (Roberson and Firestone, 1992).

There are evidences that xanthan stabilizes soil against disruptive effect of wetting and drying cycles (Czarnes et al., 2000). In comparison with control soil and dextran, soils amended with xanthan were less sensitive to this kind of stress. Differences in structure of both polysaccharides could explain their different behaviors. Rosenzweig et al. (2012) also tested the WHC of two sandy soils amended with xanthan, and observed that the addition of >1% xanthan increased dramatically the water holding capacity of the soil, as well as soil porosity.

Many of the studies that evaluate the application of microbial biopolymers in soil are in the engineering area. There are several studies that evaluate the application of microbial biopolymers and plant polymers, such as guar gum and cellulose for stabilization and soil binding for constructions. Such studies in the engineering area also confirm the usefulness of biopolymers application in soil, but with different purposes.

The strength of biopolymers can be observed by their application in the fields of construction and geotechnical engineering, as soil binders (Chang et al., 2017). The commercial polymer from *Aureobasidium pullulans* was efficient in the treatment and stabilization of a residual Korean soil, increasing the compressive strength of soil more than 200% (Chang and Cho, 2012). It was considered an economically competitive and environmentally friendly alternative for soil binding. In another study, a very small amount of microbial EPS (such as xanthan and gellan gum 0.5%) mixed with soil resulted in a higher compression strength in comparison to the addition of a large amount of cement. Xanthan forms connection bridges between particles, thereby enhancing particle alignment and improving strength. The effect is a result of the matrix strength and electrostatic bonds between xanthan and fine soil particles. These polymers can be naturally decomposed, not requiring construction demolition. They are promising for construction as building materials (Chang et al., 2015). The application of xanthan gum can also be used to treatment of collapsible soil, reducing collapsible potential (Ayeldeen et al., 2017).

In addition to the direct application of EPS to soil, there are evidences that EPS production in soil can be modulated by N management. Roberson et al. (1995) evaluated the effect of the N addition in EPS production and soil aggregation, by indirect measurements, carbohydrate content, and monosaccharide composition. While intermediate and high amount of N fertilization gave similar crop yield, the soil properties had different results. Intermediate N fertilization induced better aggregation and saturated hydraulic conductivity, and the monosaccharide composition was more related to microbial polysaccharide. Therefore, the addition of nutrients could also induce EPS production directly in soil, consequently improving soil aggregation.

## Conclusion and Perspectives

Microorganisms have developed several approaches to survive environmental conditions, especially in soils. EPS production is an important strategy for providing a moist environment, entrapping nutrients, facilitating chemical reactions, and protecting cells against environmental conditions, antibiotics, and attack by predators. Microbial extracellular polymers are highly diverse compounds with multiple functions that depend on their composition and structure.

Many studies have demonstrated that EPS production can increase soil aggregation, improve soil quality, and contribute to soil fertility. Moreover, in addition to improving soil structure, the presence of EPS in soil and in plant roots can improve nutrient uptake and water availability for both plants and microorganisms, thus benefiting not only the producer but also the environment as a whole. Microorganisms have an enormous potential, which can be enhanced by the improvement of the knowledge of the structure of EPS, as well as the development of microbial consortia and large scale EPS production.

Extracellular polymeric substances have long been of interest due to their biodegradability, biocompatibility, and thickening, gelling, and emulsion capacities. The polymers and their production can be manipulated to achieve high yields, but such manipulations are dependent on the characterization and physiological study of EPS-producing microorganisms. Improving polymer production requires an understanding of the underlying mechanisms and regulatory pathways. In contrast to the intensive work focused on improving EPS yield and altering the characteristics of well-known polymers, novel EPS and polymers produced by less studied microbial strains are still underexplored. The investigation of the genetic mechanisms involved in the biosynthesis of any type of molecule involves complex and time-consuming techniques, and thus, the development of knowledge in this area may proceed slowly. Many microorganisms produce EPS, and because each polymer is different, many opportunities remain for investigation and discovery.

Extracellular polymeric substances are complex substances and our understanding of their composition, structures, functions, and genetic regulation, although very broad, is far from complete. There is a need for a fundamental understanding of the genes and mechanisms involved in the biosynthesis and regulation of EPS. Furthermore, the discovery and characterization of new polymers could lead to interesting other applications, especially for the environment. EPS can be employed in wastewater treatment, recovery of polluted environments, and, potentially, in the recovery of soil aggregation and improvement of soil fertility. Advances in modern techniques and approaches, such as high-throughput sequencing, CLSM, nuclear magnetic resonance, scanning electronic microscopy, SIP in association with classic microbiology techniques will enhance efforts to discover and characterize new EPS and their functions in the soil ecosystem. The understanding of structure and properties of EPS is fundamental for understanding their interactions with soil. The combination of classic microbiology techniques with modern high-throughput methods and integration of different fields are fundamental for increasing knowledge on EPS composition, structure, function, and applications.

## Author Contributions

OC wrote the manuscript. EK and JR critically reviewed the manuscript. EK approved the final version of the manuscript.

## Conflict of Interest Statement

The authors declare that the research was conducted in the absence of any commercial or financial relationships that could be construed as a potential conflict of interest.
